# Exploring the Insomnia–Ischemic Stroke Nexus: A Comprehensive Review

**DOI:** 10.3390/jcm13061622

**Published:** 2024-03-12

**Authors:** Andreia Matas, Nuno Pinto, Bebiana Conde, Maria Vaz Patto

**Affiliations:** 1 Faculty of Health Sciences, Universidade da Beira Interior, 6201-001 Covilhã, Portugal; nfcpinto@gmail.com (N.P.); mariavazpato@gmail.com (M.V.P.); 2 Neurology Department, Centro Hospitalar Trás-os-Montes E Alto Douro, 5000-508 Vila Real, Portugal; 3 CICS-UBI—Health Sciences Research Centre, University of Beira Interior, 6201-001 Covilhã, Portugal; 4 Pulmonology Department, Centro Hospitalar Trás-os-Montes E Alto Douro, 5000-508 Vila Real, Portugal; bebianaconde@gmail.com

**Keywords:** insomnia, insomnia symptoms, sleep duration, cardiovascular factors ischemic stroke

## Abstract

**Background:** This study investigates the relationship between sleep patterns and ischemic stroke beyond the predominant focus on obstructive sleep apnea. Through a systematic review of the existing literature, we aim to elucidate the connections between insomnia, sleep duration, cardiovascular factors, and ischemic stroke onset. **Methods:** We searched databases, including MEDLINE, SciELO, Scopus, and Science Direct, using an adapted PICO framework. Using a search strategy with MeSH words, keywords, and expressions related to insomnia and stroke, we included clinical trials and analytical observational epidemiological studies, comprising cohort, case–control, and cross-sectional studies. **Results:** Following the initial search, we identified 984 records, with 779 titles and abstracts screened for eligibility after removing duplicates. From these, 63 full-text articles, including 5 in the data synthesis, were reviewed. Our findings highlight a significant correlation between poor sleep quality, extreme sleep durations, and a heightened risk of ischemic stroke, along with established cardiovascular risk factors, such as hypertension, hyperlipidemia, obesity, and diabetes. **Conclusions:** This systematic review offers a comprehensive analysis of ischemic stroke prevalence and its association with cardiovascular factors, such as hypertension, hyperlipidemia, obesity, and diabetes. It suggests that poor sleep quality and extreme sleep durations, particularly long ones, are linked to a heightened risk of ischemic stroke.

## 1. Introduction

Sleep is an essential component of daily functioning and is progressively acknowledged as a factor influencing long-term well-being [1]. Sleep can be affected by several conditions, and this can concur with cerebrovascular risk [2,3]. Insomnia, characterized by difficulty falling or staying asleep [4], frequently leading to daytime cognitive and motor impairments, stands as the most prevalent sleep disorder and the second-most-common mental health condition globally [4,5]. Other sleep disorders, like chronic lack of sleep, fragmented sleep, and sleep deprivation, may also be important risk factors for cardiovascular disorders [2] So far, most of the literature regarding the relationship between sleep and ischemic stroke has focused primarily on obstructive sleep apnea [5]. For sleep breathing disorders, often strongly correlated with elevated BMI and various cardiovascular, metabolic, or central nervous system issues [6], their amelioration potentially reduces daytime sleepiness, improving associated mood comorbidities like depression and anxiety [7]. However, a growing body of evidence shows that sleep disorders, including insomnia and unfavorable sleep habits, are associated with a rise in the occurrence of vascular events, such as ischemic stroke [8,9,10,11,12]. However, results regarding these associations are conflicting [12,13,14,15,16,17,18,19,20]. Moreover, the effects of sleep characteristics not related to apnea, such as sleep duration, insomnia, and chronotype (morning or evening preference), on the risk of ischemic stroke are still uncertain [5,21].

The mechanisms through which insomnia contributes to ischemic stroke risk may be multi-factorial [18], and changes in the immune system, abnormalities in the autonomic system, and changes in the hypothalamic hypophyseal axis are all being implied [5,22]. Conditions of sleep deprivation, such as sleep restriction, insomnia, and shift work, are likely to hinder cardiovascular recovery by reducing the amount of cardioprotective stable NREM sleep [23,24]. Sleep fragmentation is a common characteristic of nearly all sleep disorders, including insomnia, and is linked to increases in sympathetic activity, which in turn is associated with cardiovascular risks, including the risk of ischemic stroke [25,26]. Dysfunction of the inflammatory system associated with oxidative stress and endothelial dysfunction is seen in individuals with insomnia and can also contribute to ischemic stroke physiopathology [27].

The association between insomnia and sleep quality in the development of hypertension, obesity, hypercholesterolemia, and diabetes is also known [28,29,30,31,32,33,34,35], which leaves doubts to the role that insomnia and sleep quality may have as a single risk factor in cardiovascular problems.

Comparing studies that relate sleep disorders and cerebrovascular events can be difficult because of substantial variability in the diagnostic criteria used [36,37], use of different diagnostic procedures and instruments, lack of control for potential confounding factors, and different demographic characteristics of the studied population [15,17,38,39]. Additionally, ischemic stroke is a diverse condition, with various subtypes characterized by unique pathogenesis and pathophysiology [40]. In the comparison of these studies, one might also consider the limitations in observational studies, namely confounding and reverse causation [41].

A thorough grasp of the evidence presented in the current literature could elucidate the possible connections between insomnia, insomnia symptoms, sleep duration, and the onset of ischemic stroke, thereby providing a direction for future research endeavors in this field.

The objective of this systematic review is to identify, critically appraise, and synthesize the evidence from observational epidemiological studies, and to evaluate the existing uncertainty in relating insomnia and sleep quality to an increased risk of ischemic stroke, as follows:

- Investigating the associations between insomnia and related sleep quality and ischemic stroke.
- Evaluating insomnia and sleep quality as a single risk factor for ischemic stroke, independently of or interacting with other cardiovascular risk factors (hypertension, obesity, hyperlipidemia or hypercholesterolemia, diabetes).

## 2. Method

The review plan was officially recorded with the International Prospective Register of Systematic Reviews (PROSPERO) under registration number CRD42023393571. To ensure consistency and transparency, we adhered to the guidelines provided by the Preferred Reporting Items for Systematic Reviews and Meta-analysis (PRISMA) [42] throughout the process of selecting and reporting studies. AM, NP, and AVP conducted thorough searches, both in databases and by hand. AM and AVP worked together to screen titles and abstracts, examine full texts, and assess the quality of the studies independently. The extraction of data was carried out by AM, BC, NP, and AVP.

### 2.1. Literature Search

We utilized a search approach focused on concepts, developed in partnership with a librarian, incorporating medical subject headings (MeSH) and relevant keywords about both stroke and insomnia. This strategy was deployed across the following electronic databases: MEDLINE, Scientific Electronic Library Online (SciELO), Scopus, and Science Direct. We used specific MeSH words, keywords, and expressions related to insomnia and stroke in our search strategy. The detailed search strategies (strings) are presented in Supplementary Materials. The search was conducted to identify studies until 27 January 2023.

### 2.2. Inclusion and Exclusion Criteria

Due to the nature of this review, an adapted PICO framework was used. The criteria mentioned below were used to determine eligibility: population, outcomes secondary outcomes, and type of studies:

(a) Population

In terms of population, we selected studies in humans in any location, with no age restrictions in the searches.

(b) Outcomes

This systematic review was intended to examine potential associations between ischemic stroke and insomnia, and sleep disorders other than obstructive sleep apnea, as well as other cardiovascular risk factors, specifically hypertension, obesity, hypercholesterolemia, and diabetes (using descriptive statistics measures), and to estimate the association (HR, risk ratio, OR, 95% CIs, mean and SD) between sleep disorders other than obstructive sleep apnea factors and ischemic stroke (with different stroke subtypes).

(c) Secondary outcomes

We analyzed demographic data, ischemic stroke, insomnia and sleep duration, and cardiovascular risk factors (hypertension, hypercholesterolemia, diabetes, and obesity).

(d) Types of Studies

We incorporated primary full-text articles that were published in English-language peer-reviewed journals. Our inclusion criteria encompassed all clinical trials and analytical observational epidemiological studies, comprising cohort, case–control, and cross-sectional studies. However, we excluded narrative literature reviews, discussion papers, non-research letters, editorials, case studies, case series, and animal studies from our analysis.

(e) Due to the type of studies we intended to include, PICO intervention was also considered as variable of interest or exposure (i.e., exposure to the disease, risk behavior and/or prognostic factor). Comparison was also not chosen as an exclusion criteria.

The search results were imported into Mendeley Reference Manager, and any duplicate entries were eliminated. Two reviewers independently evaluated the relevance of titles and abstracts generated from the electronic searches. Titles and abstracts deemed irrelevant were excluded. Full-text articles that potentially met the eligibility criteria were retrieved and assessed for eligibility by the same reviewers (AM, AVP). Any discrepancies were resolved through the involvement of a third reviewer (NP). In cases where full texts were inaccessible, efforts were made to contact the authors directly for a copy. Additionally, titles specifically related to sleep, insomnia, and ischemic stroke were manually searched.

### 2.3. Data Extraction

A study-specific template was developed using Microsoft Excel software (Version 2401 (Build 17231.20236)), tested, and improved before data extraction. In cases where direct data were not available, information from figures and charts was extracted, with interpretation adjusted based on consensus between the two authors. Additionally, original article authors were contacted for clarification on any data not explicitly set out in the papers.

For each study included, we gathered the following details: citation information (such as authors, publication year, title, journal name, volume, and page numbers); demographic data of participants (including age, gender, employment status, and country of origin); study specifics (baseline and follow-up participant numbers); methodology details (study design, sampling technique, setting, characteristics of comparison groups); stroke particulars (type, severity, location, diagnostic method, recurrence, time elapsed since stroke); details regarding insomnia or insomnia symptoms (assessment method, definition, incidence, prevalence, pre-stroke sleep quality); concurrent health conditions (current or pre-existing psychological, psychiatric, or physical ailments); cardiovascular risk factors (like hypertension, hypercholesterolemia, obesity, diabetes); funding sources; and any potential conflicts of interest.

### 2.4. Study Quality

Two separate reviewers independently evaluated the quality of the studies using the Mixed Methods Appraisal Tool (MMAT), a tool designed for assessing studies included in systematic mixed studies reviews. The MMAT serves as a checklist to appraise and describe studies simultaneously. Each study and its components were assessed based on their suitability for addressing the research question, the risk of selection bias, the accuracy of exposure measurement, and the assessment of outcomes.

### 2.5. Data Analysis

Our main strategy involved delivering a narrative overview of the results, supplemented by tables outlining the particulars of the studies, including a synopsis of the findings and an evaluation of the quality of the included studies. We adhered to the PRISMA checklist to ensure proper reporting of the systematic review.

## 3. Results

**Figure 1:**
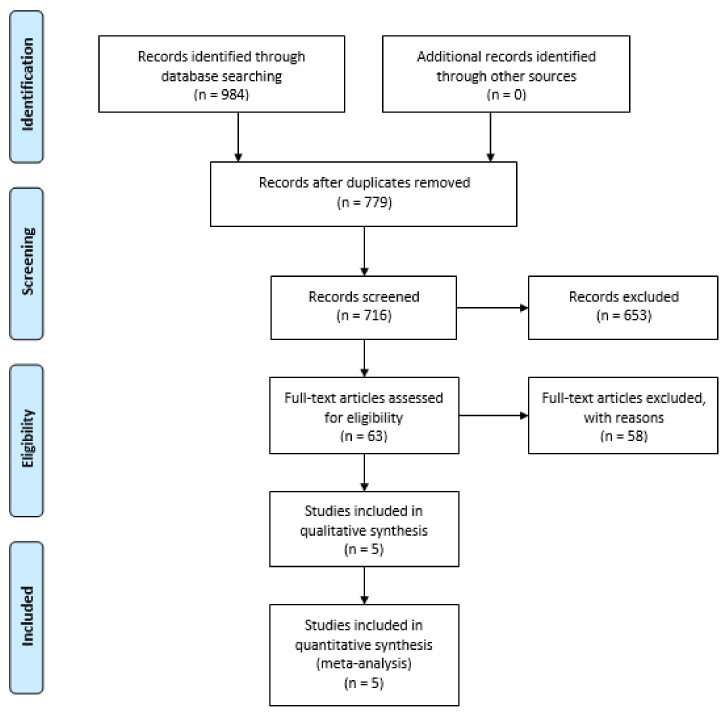
PRISMA flowchart.

We initially found 984 records via database searches. Following the elimination of duplicates, we screened 779 titles and abstracts for eligibility, excluding 716 and retaining 63 for full-text review. Subsequently, 58 were further excluded, resulting in 5 studies eligible for inclusion in the data synthesis (see Figure 1).

### 3.1. Study Characteristics

The characteristics of the included studies [43,44,45,46,47] are provided in Table 1. The identified studies were published between 2008 and 2020. Two studies were cross-sectional, and three were prospective cohort studies. These studies reported data for 620,072 participants, of which 48,206 were individuals with stroke (sample size *n* = 2846–487,200).

### 3.2. Demographic Characteristics

Only Kim et al. (2019) [44] and Pergola et al. (2017) [45] reported participants’ mean age, and the remaining three articles only reported age ranges. None of the five studies reported the mean age for individuals with stroke.

Chen et al. (2008) [46] included only women. In the other four studies, on average, 49.2% of the subjects studied were male.

### 3.3. Study Setting

Zeng et al. (2019) [43] and Kim et al. (2019) [44] carried out studies in a biobank setting, and the other three were population-based cohort studies (Pergola et al. (2017), Chen et al. (2008), and Zhou et al. (2020)) [45,46,47]. These studies include participants from North America (Kim et al. (2019), Pergola et al. (2017), and Chen et al. (2008)) [44,45,46], *n* = 3, and Asia (Zeng et al. (2019) [43] and Zhou et al. (2020) [47]), *n* = 2.

### 3.4. Insomnia and Insomnia-Related Symptoms Assessment

All five included studies used a sleep questionnaire to assess insomnia and/or insomnia-related symptoms (Table 2). None of the included studies included specific diagnostic tools in the assessment of insomnia and insomnia-related symptoms.

Zeng et al. (2019) [43] established the categories “difficulties in initiating sleep or maintaining sleep” (11.3%) and “early morning awakening” (10.4%). Pergola et al. (2017), Chen et al. (2008), Kim (2019), and Zhou et al. (2020) [44,45,46,47] assessed sleep duration, and Zhou et al. (2020) [47] additionally assessed midday napping and sleep quality.

### 3.5. Stroke Assessment

Kim et at (2019), Pergola et al. (2017), and Chen et al. (2008) [44,45,46] used clinical data to diagnose stroke, and Zeng et al. (2019) and Zhou et al. (2020) [43,47] used the International Classification of Diseases 10th Revision (ICD-10) to diagnose stroke.

### 3.6. Insomnia/Insomnia Symptoms Preceding Stroke

Zeng et al. (2019) [43] reported a prevalence of 16.4% of insomnia symptoms in the population.

The remaining four studies assessed the duration of sleep in the studied population, with short sleep duration present and ranging between 8.3 and 39.0% of the subjects, normal sleep duration ranging between 26.0 and 61.8% of the subjects, and long sleep duration ranging between 2.8 and 35.0% of the subjects.

Zhou et al. (2020) [47] also assessed daytime napping, present in 7.6% of the subjects.

### 3.7. Impact of Insomnia/Insomnia Symptoms on Stroke Incidence

Zeng et al. (2019) [43] found that individuals experiencing “difficulties in initiating or maintaining sleep”, “early morning awakening”, and “daytime dysfunction” exhibited slightly elevated risks of developing ischemic stroke.

Kim et al. (2019) and Pergola et al. (2017) [44,45] reported that those with extremely short and extremely long sleep durations had higher all-cause mortality (cardiovascular mortality included) compared to those with normal sleep durations.

According to Chen et al. (2008) [46], the negative impact of prolonged sleep appears to be separate from the heightened risk of ischemic stroke linked to frequent snoring and daytime sleepiness.

In terms of sleep patterns, Zhou et al. (2020) [47] observed that individuals experiencing poor sleep quality were more susceptible to ischemic stroke. Moreover, the study highlighted notable combined effects, such as the combination of extended sleep duration with midday napping, and extended sleep duration with poor sleep quality, on the overall risk of stroke.

### 3.8. Stroke Characteristic

Stroke type was reported in detail in two of the five studies (Chen et al. (2008) and Zhou et al. (2020)) [46,47]. Three studies did not provide a detailed stroke subtype. Chen et al. (2008) [46] established the subtypes of “ischemic” and “non-ischemic stroke”. Zhou et al. (2020) [47] differentiated between ischemic and hemorrhagic stroke types and further categorized ischemic strokes into subtypes, including large-artery occlusive infarction, lacunar infarction, cardioembolic infarction, and other demonstrated causes of infarction, based on the Trial of Org 10172 in Acute Stroke Treatment classification [48].

With regards to lesion location, none of the included studies described the topography of the ischemic strokes in the included population.

None of the studies reported the time between insomnia and insomnia symptom onset and the occurrence of ischemic stroke.

### 3.9. Association between Sleep Problems and Cardiovascular Risk Factors

All five included studies presented data regarding body mass index (BMI), hypertension, hyperlipidemia, and diabetes. Four of the studies also presented data regarding hyperlipidemia.

#### Body Mass Index (BMI)

Kim et al. (2019) [44] observed that individuals with a short sleep duration (<6.5 h) had a BMI of 31 ± 7 kg/m^2^, those with normal sleep duration (≥6.5 to <7.5 h) had a BMI of 30 ± 6 kg/m^2^, and those with long sleep duration (≥7 h) had a BMI of 29 ± 6 kg/m^2^. Additionally, Zhen et al. (2019) found that individuals experiencing difficulties in initiating or maintaining sleep, early morning awakening, or daytime dysfunction had an average BMI of 32.3 kg/m^2^.

Chen et al. (2008) [46] found a trend of BMI distribution across different sleep durations: 9.3% of individuals sleeping ≤5 h/night, 26.4% of those sleeping 6 h/night, 37.5% of those sleeping 7 h/night, 21.8% of those sleeping 8 h/night, 4.5% of those sleeping 9 h/night, and 0.5% of those sleeping ≥10 h/night were underweight (BMI < 18.5 kg/m^2^). Additionally, 6.6% sleeping ≤5 h/night, 25.6% sleeping 6 h/night, 40.3% sleeping 7 h/night, 23.3% sleeping 8 h/night, 3.8% sleeping 9 h/night, and 0.4% sleeping ≥10 h/night had a normal weight (BMI 18.5–24.9 kg/m^2^). Moreover, 5.5% of those sleeping ≤5 h/night, 16.3% sleeping 6 h/night, 20.8% sleeping 7 h/night, 13.1% sleeping 8 h/night, 2.4% sleeping 9 h/night, and 0.4% sleeping ≥10 h/night were overweight or obese (BMI ≥ 25 kg/m^2^).

Zhou et al. (2020) [47] reported varying BMIs among different sleep durations: 24.4 kg/m^2^ for those sleeping <6 h/night, 24.6 kg/m^2^ for 6 to <7 h/night, 24.3 kg/m^2^ for 7 to <8 h/night, 24.2 kg/m^2^ for 8 to <9 h/night, and 24.0 kg/m^2^ for those sleeping ≤9 h/night.

Pergola et al. (2017) [45] found that 73.8% of participants fell under the categories of underweight, normal weight, or overweight, with 26.2% classified as obese. However, the study did not provide a BMI characterization based on different sleep durations.

### 3.10. Hypertension

Hypertension prevalence varied considerably across studies, ranging from 10.3% to 87%.

Kim et al. (2019) [44] reported that individuals with a short sleep duration (<6.5 h) had the highest hypertension prevalence at 87%, followed by 84% for normal sleep duration (≥6.5 to <7.5 h), and 85% for long sleep duration (≥7.5 h).

Zhen et al. (2019) [43] found that approximately 33% of individuals experiencing various sleep disturbances had hypertension.

In contrast, Chen et al. (2008) [46] found the hypertension prevalence to be 7.5%, 26.4%, 38.9%, 22.8%, 3.9%, and 0.5% among different sleep duration groups.

Zhou et al. (2020) [47] noted hypertension rates ranging from 64.7% to 69.2% across different sleep durations.

Pergola et al. (2017) [45] identified 31.2% of participants as hypertensive, although they did not characterize hypertension prevalence across sleep duration subgroups.

### 3.11. Diabetes Mellitus

Diabetes prevalence varied widely across studies, ranging from 5.7% to 40%.

Kim et al. (2019) [44] reported the highest prevalence among individuals with a short sleep duration (<6.5 h) at 40%, followed by 35% for normal sleep duration (≥ 6.5 to <7.5 h) and 37% for long sleep duration (≥7.5 h).

Zhen et al. (2019) [43] found that approximately 6–7% of individuals experiencing different sleep disturbances had diabetes.

Chen et al. (2008) [46] observed diabetes prevalences of 15.2%, 29.2%, 31.0%, 19.3%, 4.3%, and 0.9% across various sleep duration groups. Zhou et al. (2020) noted diabetes rates ranging from 14.1% to 16.4% across different sleep durations.

Pergola et al. (2017) [45] identified 10.0% of participants as diabetic, but they did not characterize diabetes prevalence across sleep duration subgroups.

### 3.12. Hyperlipidemia

Hyperlipidemia prevalence ranged from 9.7% to 78% across studies.

Kim et al. (2019) [44] reported 75%, 72%, and 78% prevalence among individuals with short, normal, and long sleep durations, respectively.

Zhen et al. (2019) [43] did not provide specific data on hyperlipidemia.

Chen et al. (2008) [46] observed a correlation between sleep duration and diabetes prevalence. They found that diabetes rates were 15.2%, 29.2%, 31.0%, 19.3%, 4.3%, and 0.9% among individuals with different sleep durations, ≤5 h, 6 h, 7 h, 8 h, 9 h, and ≥10 h, respectively.

Zhou et al. (2020) [47] found hyperlipidemia rates ranging from 38.9% to 44.6% across various sleep durations.

Pergola et al. (2017) [45] did not characterize hyperlipidemia prevalence across sleep duration subgroups.

### 3.13. Other Covariates

Data on current drinking were reported in three studies, with prevalence rates ranging from 6.9% to 76%, while two studies provided information on this topic, showing rates between 14.1% and 30.4%.

Only one study addressed regular exercise, reporting a prevalence of 74.4%, along with symptoms of anxiety (2.0%). Additionally, three studies included data on depression prevalence, which varied from 8.7% to 17.6%.

### 3.14. Mortality

Kim et al. (2019) [44] was the sole study to provide mortality data, revealing a mortality rate of 15%. Among all deaths, 2% were attributed to stroke, distributed as follows: 2% for individuals with short sleep duration (<6.5 h), 2% for those with normal sleep duration (≥6.5 to <7.5 h), and 3% for individuals with long sleep duration (≥7.5 h). None of the remaining four studies included mortality data.

### 3.15. Critical Appraisal

In terms of the methodological quality of the studies, all five included studies presented good quality. A summary of quality ratings is provided in Table 3.

## 4. Discussion

To our knowledge, there has been no systematic review examining the connections between ischemic stroke, sleep issues, and cardiovascular risk factors, like hypertension, hypercholesterolemia, diabetes, and obesity. Our findings indicate that research in this area varies significantly across studies, limiting generalizations. Some studies, such as those by Zheng (2019), Zhou (2020), and Chen (2008) [43,45,47], isolated insomnia and sleep symptoms as singular variables, while others like Kim (2019) and Pergola (2017) [44,45] linked insomnia and sleep symptoms to vascular risk factors.

Zheng et al. (2019) [43] conducted a prospective cohort study involving 487,200 participants from ten regions in China, finding that individual insomnia symptoms were independent risk factors for cerebral vascular diseases, ischemic heart disease, and ischemic stroke, particularly among younger adults or those without hypertension. Chen et al. (2008) [46] studied 93,175 postmenopausal women, discovering that long sleep duration was associated with an increased risk of ischemic stroke. Kim et al. (2019) and Pergola et al. (2017) [44,45] both examined insomnia and sleep symptoms about vascular risk factors, with Kim et al. finding that a short sleep duration was associated with higher cardiovascular mortality, including from ischemic stroke.

Data from various studies suggest that sleep patterns and quality play significant roles in ischemic stroke risk. Insomnia and poor sleep quality appear to elevate the risk of cerebral vascular disease, while both short and long sleep durations carry risks. However, the profiles of increased stroke risk in individuals with insomnia and sleep symptoms remain complex due to heterogeneous data across studies.

Some work was already performed trying to connect sleep disturbances and stroke. Silva et al. (2022) conducted a systematic review of four studies to investigate the link between chronic insomnia disorder and stroke risk in adults [49]. However, due to variations in how insomnia was classified across the studies and inadequate data, it was inconclusive whether chronic insomnia disorder was a risk factor for stroke. In contrast, Wu et al. (2023) compiled 25 meta-analyses of prospective cohort studies, presenting 63 summary relative risk estimates for 29 distinct outcomes [50] They revealed that insomnia is linked to various adverse outcomes, particularly cardiovascular issues and mental disorders. Nevertheless, the authors noted a lack of high-quality evidence supporting the association between insomnia and health outcomes. Shiyu Hu et al. (2021) conducted a meta-analysis exploring the connection between specific insomnia symptoms and cardiovascular disease risk [51]. They discovered that insomnia symptoms, such as difficulty initiating or maintaining sleep, non-restorative sleep, and early morning awakening, were linked to an increased incidence of cardiovascular disease in individuals without prior cardiovascular issues. However, the meta-analysis focused solely on studies with well-defined insomnia symptoms, potentially overlooking relevant data that could elucidate associations between individual insomnia symptoms and cardiovascular disease risk.

Considering the bidirectional relationship between insomnia, sleep, and cardiovascular health, post-stroke insomnia should also be of significant concern. With approximately one-third of stroke survivors meeting the diagnostic criteria for insomnia, which rises to four in ten when considering symptoms alone, there is also a need for routine screening of sleep disorders post-stroke, particularly targeting insomnia and employing appropriate diagnostic tools [52].

The exclusion of several studies from our analysis underscores the importance of recognizing cardiovascular risk factors, particularly in the context of stroke and sleep disorders. The diverse methodological approaches, along with variations in insomnia definitions and diagnostic tools employed across studies, introduce significant limitations to the conclusions drawn from this review. Nevertheless, our review provides a comprehensive examination of the interplay between sleep and cardiovascular issues, illuminating their potential co-occurrence, particularly concerning conditions like stroke. To address heterogeneity and limitations more thoroughly, future research should focus on standardizing methodologies and definitions, thereby enhancing comparability across studies. Additionally, it is essential to acknowledge the tentative nature of our results and emphasize the need for further investigations to elucidate the complex relationship between sleep disorders and cardiovascular health.

## 5. Conclusions

The studies included in our systematic review were heterogeneous regarding the evaluation tools used in the assessment of stroke, insomnia, and sleep disturbances.

Still, this systematic review provides a comprehensive overview of the prevalence of ischemic stroke and several cardiovascular factors (hypertension, hyperlipidemia, obesity, and diabetes), indicating that poor sleep quality is associated with a higher risk of ischemic stroke, as is an extremely short and extremely long sleep duration, with an apparent accentuation on the latter parameter.

Our review did not allow us to extract data on the potential associations between insomnia and other sleep disturbances, including sleep quality and duration, and the development of vascular risk factors.

Additional research with broader and well-designed studies is needed to clarify the potential associations between insomnia and sleep disorders with incident ischemic stroke as well other cardiovascular risk factors.

## Figures and Tables

**Table 1:** Study characteristics.

Study	Country	Total N (Population)	Total N (%, n of Stroke)	Total N (%, n of Insomnia/Sleep Disturbance)	Design	Sampling	Mean Age, Years (SD)	Gender, % Male	Setting	Stroke Types	Follow Up, Years	Mortality
Bang Zheng et al. (2019) [43]	China	487,200	45,316 (9.3%)	7431 (16.4%) insomnia symptoms	Prospective cohort study	Consecutive	30 to 79 yo	199,241 (40.9%)	China Kadoorie Biobank—recruited participants from 10 areas across China	NR	Median 9.6 y	NR
Jeong Hwan Kim et al. (2019) [44]	USA	2846	68 (2%)	*N* = 1110 (39%) <6.5 h (short SD), *N* = 740 (26%), ≥6.5 h to <7.5 h (normal SD), and *N* = 996 (35%) ≥7.5 h (long SD)	Cross-sectional study	Consecutive	64 yo ± 13	1775 (62.0%)	Emory Cardiovascular Biobank	NR	Median 2.8 y	412 (15%)
Brianna L. Pergola et al. (2017) [45]	USA	5101	218 (2.9%)	61.8% slept 7–9 h on average, 31.5% slept 5–6 h, 3.8% slept 1–4 h, and 2.8% slept for 10–18 h	Cross-sectional, population-based study	Random digit dialed telephone survey	18–34 yo: *N* = 895 (30.4%); 35–44 yo: *N* = 631 (17.6%); 45–64 yo: *N* = 1915 (33.9%); 65 yo: ˃65 yo *N* = 1607 (18.1%)	2195 (50.2%)	2013 Nevada Behavioral Risk Factor Surveillance System	NR	NA	NR
Jiu-Chiuan Chen et al. (2008) [46]	USA	93,175	1166 (1.25%)	≃8.3% short SD ≤ 5 h/night, 4.6% long sleepers (≥9 h/night)	Prospective study	Consecutive	50–79 yo	0%	Women’s Health Initiative Observational Study cohort	IS and non-IS	Average of 7.5 y	NR
Lue Zhou et al. (2020) [47]	China	31,750	1151 definite IS; 287 probable IS (*N* = 1438 strokes, 4.5%)	23.9% SD ≥ 9 h/night; 7.6% midday napping >90 min	Prospective cohort study	Consecutive	Average 61.7 yo	13,996 (44.1%)	Dongfeng-Tongji cohort	IS, including subtypes *	Average 6.2 ± 2.4 y	NR

SD: Sleep duration; IS: Ischemic stroke; NR: Not reported; NA: Not applicable. * Large-artery occlusive infarction, lacunar infarction, cardioembolic infarction, and other demonstrated causes of infarction according to the Trial of Org 10172 in Acute Stroke Treatment classification.

**Table 2:** Diagnostic tools to assess stroke and sleep disturbances.

Study	Stroke Assessment	Insomnia/Other Sleep Disturbances Assessment	Subtype of Insomnia	Insomnia/Sleep Disturbance Overall Prevalence %	Development of Stroke Demographic Characteristics %, Mean (σ)	Other Sleep Characteristics Demographic Characteristics/Comorbidity
Bang Zheng et al. (2019) [43]	ICD-10 [stroke (I60–I61; I63–I64); IS (I63)]	Questionnaire—specific insomnia symptoms for at least 3 d/wk in the past month	DIMS, EMA, and DD	DIMS 55127 (11.3%); EMA 50691 (10.4%)	Associations of DD with the incidence of IS and its subtypes were Identified only in male participants, although the sex heterogeneity was not statistically significant	The associations of DIMS, EMA, and DD with total CVD incidence were consistently stronger in younger participants
Jeong Hwan Kim et al. (2019) [44]	Clinical diagnosis	Sleep questionnaire—“How many hours of sleep do you usually get each night (or when you usually sleep)?”	NA	NA	NR	Subjects reporting short SD tended to be younger, ♀, and black, with higher BMI, DM, and HF compared to those with normal SD. Those with long SD were older and more likely to have a history of hyperlipidemia, smoking, and prior MI. Independent predictors of short SD were ♀ sex, black race, and higher BMI, while older age and history of smoking were associated with long SD
Brianna L. Pergola et al. (2017) [45]	Clinical diagnosis	Standardized core questionnaire, along with optional modules, and state-added questions	NA	NA	After adjusting for age, sex, race, marital status, education, and insurance, ♂ were found to be 1.34 times more likely to report a IS compared to ♀, although this did not reach statistical significance. Individuals aged 18–34 yo were 95.8% less likely to report a IS compared to those aged 65 yo and older after adjusting for gender, age, race, marital status, education, and insurance. Those aged 35–44 yo and 45–64 yo were 78% and 59.1% less likely, respectively, than individuals 65 yo and older to have a IS (*p*-value < 0.05 for both)	Hypertensive individuals had a 3.67-fold higher likelihood of reporting stroke compared to those with normal blood pressure (*p* < 0.0001). High cholesterol was associated with a 2.32-fold increased likelihood of stroke (*p* < 0.0001). Diabetic individuals were nearly twice as likely to report a cardiovascular condition (*p* = 0.003), and those with depressive disorder had a 1.8-fold higher likelihood of a cardiovascular condition compared to those without (*p* < 0.05)
Jiu-Chiuan Chen et al. (2008) [46]	Clinical diagnosis	Questionnaire—“hours of sleep on a typical night during the past 4 weeks” (≤5, 6, 7, 8, 9, ≥10)	NA	Approximately 8.3% reported short SD ≤ 5 h/night, while 4.6% were long sleepers (≥9 h/night)	After adjusting for age and race, there was a 19% (95% CI: 3–37%) increase in RR in with ≤ 6 h/night of sleep. After further adjustment for socioeconomic status, depressive symptoms, HT use, and conventional CVD risk factors, the increased RR associated with ≤ 6 h/night of sleep became statistically non-significant. In contrast, there was a consistent and graded increase in RR observed for ♀ with 8 h/night of sleep and those with ≥9 h/night, with RRs increasing approximately by 25% among women with 8 h/night of sleep and by 70% in those with ≥9 h/night of sleep across all adjusted models.	Participants with higher BMI above the normal range were more likely to experience short DS, while current users of HT medication were less likely to report short SD. Those with existing coronary heart disease/cardiovascular disease, treated DM, hypertension, hypercholesterolemia, or depression had a higher likelihood of reporting short SD. ♀ with similar conditions were more likely to experience long SD. Individuals reporting longer SD or extended MN were more likely to be ♂, less educated, current smokers, current drinkers, and physically inactive. Longer SD was associated with a lower likelihood of hypertension, while extended MN was linked to a higher likelihood of hypertension, diabetes, and hyperlipidemia
Lue Zhou et al. (2020) [47]	ICD-10 codes I60–I61, I63–I64, I69.0–I69.1, and I69.3–I69.4	Self-administrated questionnaire (SD, MN, and sleep quality)	NA	23.9% slept ≥ 9 h/night and 7.6% reported MN > 90 min	The association of total stroke with long SD seemed to be more evident among individuals who were ≥65 yo, or with hypertension or hyperlipidemia or DM, but no interaction was observed except for DM (*p* for interaction = 0.033). Similarly, the risk of total stroke with long MN appeared to be more pronounced among persons who were overweight, but no interaction was found	Participants who reported SD ≥ 9 h/night or having MN ≥ 90 min were more likely to be ♂ less educated, current smokers, current drinkers, and physically inactive (all *p* < 0.05). Those reporting SD ≥ 9 h/night were less likely to have hypertension, whereas individuals having MN ≥ 90 min were more likely to have hypertension, DM, and hyperlipidemia compared to reference groups.

IS: Ischemic stroke; SD: Sleep duration; DIMS: Difficulties in initiating or maintaining sleep; EMA: Early morning awakening; DD: Daytime dysfunction; CVD: Cardiovascular diseases; MN: Midday napping; BMI: Body mass index; DM: Diabetes Mellitus; HF: Heart failure; MI: Myocardial infarction; RR: Relative risk; HT: Hypertensive; NR: Not reported; NA: Not applicable.

**Table 3:** Study quality appreciation according to the Mixed Methods Appraisal Tool (MMAT), version 2018.

		Screening Questions		Qualitative Studies				
First Author	Year	Are There Clear Research Questions?	Does the Collected Data Allow Us to Address the Research Questions?	Is the Qualitative Approach Appropriate to Answer the Research Question?	Are the Qualitative Data Collection Methods Adequate to Address the Research Question?	Are the Findings Adequately Derived from the Data?	Is the Interpretation of Results Sufficiently Substantiated by Data?	Is There Coherence between Qualitative Data Sources, Collection, Analysis, and Interpretation?
Bang Zheng	2019	Yes	Yes	Yes	Yes	Yes	Yes	Yes
Jiu-Chiuan	2008	Yes	Yes	Yes	Yes	Yes	Yes	Yes
Jeong Hwan Kim	2019	Yes	Yes	Yes	Yes	Yes	Yes	Yes
Brianna L. Pergola	2017	Yes	Yes	Yes	Yes	Yes	Yes	Yes
Lue Zhou	2020	Yes	Yes	Yes	Yes	Yes	Yes	Yes

## Supplementary Materials

The following supporting information can be downloaded at: https://www.mdpi.com/article/10.3390/jcm13061622/s1, The Supplementary Materials comprise the research strategy and search strings utilized in the databases PUBMED/MEDLINE, Scielo, Scopus, and Science Direct for this systematic literature review.
