# Supplementary material for: Exploring the Insomnia–Ischemic Stroke Nexus: A Comprehensive Review
Source: J Clin Med. 2024 Mar 12;13(6):1622. doi: 10.3390/jcm13061622 (PMC10970733; doi:10.3390/jcm13061622)
Supplement: Supplementary file 1 [file jcm-13-01622-s001.zip › jcm-2891045-supplementary.pdf]

Search strategy

String PUBMED/MEDLINE

("ischaemic stroke"[All Fields] OR "Ischemic Stroke"[All Fields] OR "intracranial embolism"[All Fields] OR "thrombotic stroke"[All Fields] OR "thrombosis"[All Fields] OR "lacunar stroke"[All Fields] OR ("Ischemic Stroke"[MeSH Terms] OR "Brain Infarction"[MeSH Terms])) AND ("insomnia"[All Fields] OR "lack of sleep"[All Fields] OR "poor sleep quality"[All Fields] OR "late sleep onset"[All Fields] OR "sleeplessness"[All Fields] OR "insomn*"[All Fields] OR "Sleep Initiation and Maintenance Disorders"[MeSH Terms])

String Scielo

(("ischaemic stroke" OR "Ischemic Stroke" OR "intracranial embolism" OR "thrombotic stroke" OR "lacunar stroke" OR "Brain Infarction")) AND (("insomnia" OR "lack of sleep" OR "poor sleep quality" OR "late sleep onset" OR "sleeplessness" OR "insomn*" OR "Sleep Initiation and Maintenance Disorders"))

String Scopus

(TITLE-ABS-KEY (("ischaemic stroke" OR "Ischemic Stroke" OR "intracranial embolism" OR "thrombotic stroke" OR "lacunar stroke" OR "Brain Infarction")) AND TITLE-ABS-KEY (("insomnia" OR "lack of sleep" OR "poor sleep quality" OR "late sleep onset" OR "sleeplessness" OR "insomn*" OR "Sleep Initiation and Maintenance

String Science Direct

("ischaemic stroke" OR "Ischemic Stroke" OR "intracranial embolism" OR

"thrombotic stroke" OR "Brain Infarction") AND ("insomnia" OR "lack of sleep" OR "poor sleep quality" OR "late sleep onset")
